# Diet across the Lifespan and the Association with Breast Density in Adulthood

**DOI:** 10.1155/2013/808317

**Published:** 2013-02-02

**Authors:** Jessica Lindgren, Joanne Dorgan, Jennifer Savage-Williams, Donna Coffman, Terryl Hartman

**Affiliations:** ^1^Department of Nutritional Sciences, The Pennsylvania State University, 110 Chandlee Laboratory, University Park, PA 16802, USA; ^2^Fox Chase Cancer Center, 333 Cottman Avenue, Philadelphia, PA 19111, USA; ^3^Department of Nutritional Sciences, Center for Childhood Obesity Research, The Pennsylvania State University, 129 Noll Laboratory, University Park, PA 16802, USA; ^4^The Methodology Center, The Pennsylvania State University, 400 Calder Square II, State College, PA 16801, USA

## Abstract

Studies have shown inconsistent results regarding the association between dietary factors across the lifespan and breast density and breast cancer in women. Breast density is a strong risk factor for breast cancer, and the mechanism through which it influences cancer risk remains unclear. Breast density has been shown to be modifiable, potentially through dietary modifications. The goal of this paper is to summarize the current studies on diet and diet-related factors across all ages, determine which dietary factors show the strongest association with breast density, the most critical age of exposure, and identify future directions. We identified 28 studies, many of which are cross-sectional, and found that the strongest associations are among vitamin D, calcium, dietary fat, and alcohol in premenopausal women. Longitudinal studies with repeated dietary measures as well as the examination of overall diet over time are needed to confirm these findings.

## 1. Introduction

 Breast cancer (BC) is the most commonly diagnosed cancer and the second leading cause of cancer death among women [1]. Alcohol consumption, physical activity, elevated after menopausal body mass index (BMI) [2], age at menarche and menopause [3], and family history and genetic mutations [4] are a few of the well-established BC risk factors. In addition, breast density (BD), or the amount of dense fibroglandular tissue present in the breast, has been related to BC risk; women who have breast densities of 75% or more have up to a 4-5-fold increase in BC risk [5]. Consequently, BD is often thought of as an intermediate on the BC development continuum that can be measured, assessed, and targeted for potential cancer prevention strategies [5–8]. Even so, little is known about the mechanism through which BD may affect breast cancer risk [9]. Breast tissue develops mostly during puberty and continues to undergo changes throughout several life stage events, such as pregnancy [3, 10, 11]. This paper will examine research on diet and diet-related factors captured across the lifespan and the association with adult BD.

## 2. Methods 

**Figure 1:**
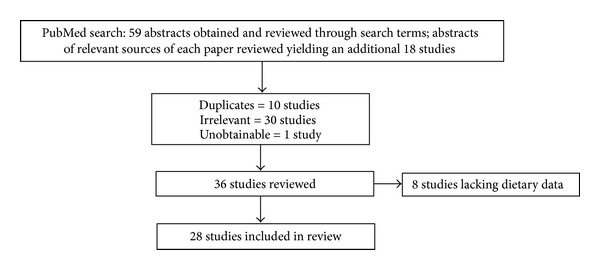
Lindgren, Dorgan, Savage-Williams, Coffman, and Hartman “Diet across the Lifespan and the Association with Breast Density in Adulthood.”

 A literature search of the PubMed database of the United States National Library of Medicine was conducted to find human studies that evaluated the associations between BD measures and diet isn the form of either single nutrients or whole dietary patterns. Both observational and diet intervention studies conducted at any stage of the lifespan were considered. Observational studies were included if they had recorded individual's dietary intake of foods or energy with dietary assessment tools such as a dietary recall (DR), food frequency questionnaire (FFQ), food record (FR), or other relevant assessment tools. Relevant studies were identified using the following search terms in multiple combinations: “adolescent diet and breast density,” “diet and breast density,” “childhood and breast density,” “diet and parenchymal patterns” and “mammographic breast density and diet.” The search was limited to full-text publications written in English. As illustrated in Figure 1, a total of 77 studies were identified. After all exclusions, 28 studies were included in this paper. 

### 2.1. Measurement of Breast Density

BD can be measured two (2D) and three dimensionally (3D), with the most common being through 2D mammography. Mammography measures the area of dense tissue (ADT) and the total area of the breast. Percent dense area (PDA) is often reported and is estimated as the proportion of dense fibroglandular tissue area to total breast area [9]. Area of nondense tissue (ANDT), which is primarily adipose tissue, also can be estimated. Magnetic resonance imaging (MRI) and ultrasound also are used to measure BD. These 3D modalities measure volume of dense tissue (VDT) and percent dense volume (PDV). Percent densities measured by mammography and MRI are highly correlated in the general population and among women who have low breast densities (*r* = 0.73) [38], but this correlation is attenuated among women with higher mammographic density greater than 50 percent (*r* = 0.26) [38]. In addition to quantitative measures of PDA and ADT, semiquantitative and qualitative measures are frequently reported. Either the Wolfe classification, which has been further classified into Tabár, or the breast imaging-reporting and data system (BI-RADS) classification is used [39–41]. These measures often classify the breast on a four- to five-level scale ranging from low-to-high levels of fibroglandular tissue. While all methods are able to assess BD, quantitative methods provide more consistent results and a larger gradient of risk. Qualitative measures often have intervals that are too large (fewer categories) and do not capture true risk gradients [42].

### 2.2. Nondietary Factors That Influence Breast Density

In general, PDA is higher in premenopausal women compared to postmenopausal women as well as in postmenopausal women who use hormone replacement therapy (HRT), and in both pre- and postmenopausal women with a lower BMI; and is lower in women who are parous, experience their first birth at a younger age, or are smokers [9, 43, 44]. Correlates of ADT are less well-studied, but in one study the ADT was inversely associated with age and BMI [44]. The nondense compartment of the breast is adipose tissue, and higher adiposity, frequently measured by BMI, attenuates the ratio of dense tissue area to total breast area. Other characteristics associated with BD may influence estrogen, insulin-like growth factor-I (IGF-I), or insulin-like growth factor binding proteins (IGBPBs) that affect fibroglandular tissue proliferation [15, 45]. Alternatively, some characteristics, such as parity, could have direct effects on breast morphology which are reflected in PDA. 

### 2.3. Dietary Factors

This review will focus primarily on diet and diet-related factors and their potential effects on both PDA and ADT with only limited attention to endogenous risk factors that are well-studied and not modifiable. A summary of these findings can be found in Tables 10 and 11. Observational studies and clinical trials that evaluated dietary intakes during childhood, adolescence and adulthood are described.

## 3. Childhood Diet and Adult Breast Density

Much of breast development occurs during puberty; thus, factors such as childhood diet that influence the timing of puberty could potentially affect BD [46, 47]. Three studies have examined dietary habits during childhood and the effect on BD in adulthood. Mishra and colleagues [13, 14] conducted two studies in a nationally representative longitudinal British sample to examine the association of childhood diet with BD. Childhood diet was assessed at age four years by a single dietary recall completed by mothers and later linked to mammographic BD measures collected at approximately fifty years of age from pre-, peri- and postmenopausal women. After controlling for relevant confounders, the investigators observed no association between PDA and childhood calcium [14], or total energy intake or with three dietary patterns ((1) breads and fats, (2) fried potatoes and fish, and (3) milk, fruit and biscuits). A limitation of these studies is that a single dietary recall was used to assess diet, which could have contributed to the null results since multiple recalls are typically required to adequately assess usual diet [48]. Additional time points for dietary data collection, such as during adolescence, may have provided more insight into the effect of early diet on BD. 

Haars and colleagues [12] examined the association between short-term transient caloric restriction (i.e., 6–8 mos.) during the Dutch Famine (when women were aged 2–33 years) and adult BD in The Netherlands DOM-project. While this study does not necessarily fit within our inclusion criteria, it is included in this paper because of the limited data available on children. Levels of caloric restriction were retrospectively assessed through three questions regarding hunger, cold, and weight loss and categorized as absent, moderate, or severe famine exposure (FE). Degree of famine exposure at 2–9 years of age was significantly inversely associated with ANDT; mean ANDT were 77.8 cm^2^, 87.7 cm^2^ and 53.1 cm^2^ in unexposed, moderately, and severely exposed, respectively (*P*_trend_ = 0.03). Although not significant, the women who were severely energy restricted at this age also had a larger ADT and higher PDA. However, because only 15 subjects were severely restricted, results should be interpreted cautiously. 

The three studies that examined childhood diet and its effect on adult BD measures did not find associations with PDA or ADT although, in the study of the Dutch famine, severe caloric restriction early in life was significantly inversely associated with ANDT later in life [12]. In this cohort, women who were severely calorically restricted had higher levels of both IGF-1 and IGFBP-3 postmenopausally than those who were not restricted [49]. Thus, one mechanism through which caloric restriction at young ages could potentially influence adult BD may be via differential programming of the somatotrophic axis resulting in long-term effects on growth factors such as IGF-1 and IGFBP-3 that are associated with breast density [50]. However, the small sample size and indirect diet assessment limit the inferences that can be drawn from this study. Taken together, the limited data available do not provide strong support for a role of childhood diet in determining breast density, but additional large prospective studies are needed before firm conclusions can be made. 

## 4. Adolescent Diet and Adult Breast Density 

Most of breast development occurs during puberty, and diet during this time could have long-term effects on BD in adulthood. One of five studies we found a significant association between diet during adolescence and BD in adulthood [16]. In the study by Tseng et al. [16], higher red meat intakes between the ages of 12–17 years were significantly associated with increased adult PDA in 201 Chinese-American female immigrants. After adjusting for degree of acculturation and other relevant covariates, women with the highest red meat consumption were at 3 times the odds of being in the highest PDA category compared to those with the lowest red meat consumption. When stratified by menopausal status, red meat intake remained significantly positively associated in postmenopausal, but not premenopausal women. 

The remaining four studies, including 3 observational studies and one clinical trial, found no associations between dietary components or alcohol consumption during adolescence and BD in adulthood [12, 15, 17, 18]. Two studies used data from the large Minnesota Breast Cancer Family Study Cohort (MBCFSC) to examine the role of adolescent diet and alcohol consumption on BD in pre- and postmenopausal women. Diet for girls at ages 12-13 years was collected retrospectively 50 years later via a 29-item FFQ focusing on high-fat foods (e.g., meats and other animal fat sources, snacks, and desserts). Intakes of fruits, vegetables, fish, and chicken were also analyzed. In the first study, Sellers et al. [15] observed no significant associations between any of these food groups and BD in multivariate analyses stratified by menopausal status. In the second analysis, Vachon et al. [17] evaluated alcohol consumption prior to age 18 via a self-reported questionnaire collected when the majority of the women were in their sixties. “Never drinkers” had lower mean PDA than “ever drinkers” (22.2 ± 14.3% versus 26.5% ± 15.9%); however, these results were attenuated and not significant after adjustment for age, BMI, HRT use, age at first birth, and parity [17]. In the study by Haars et al. [12] described above, short-term caloric restriction in girls age 10–18 years was not associated with adult BD measures. In a clinical trial, Dorgan et al. [18] examined the long-term effects of a dietary intervention to lower fat and increase fiber intake during childhood and adolescence (the Dietary Intervention Study in Children-DISC) and observed no differences in the VDT or PDV between those participants who received the behavioral intervention and the control group [18]. Thus, similar to childhood diet, the limited data available do not provide much support for a role of adolescent diet in determining adult BD, but additional research is needed.

## 5. Adult Diet and Adult Breast Density 

The majority of studies that have evaluated associations of diet with BD assessed the effects of adult diet. A total of 26 epidemiological studies and randomized controlled trials that examined dietary intake and BD among adult women are included in this paper. 

### 5.1. Total Energy

 Three studies examined the association of total energy intake in adulthood with BD measures (Table 9). In a nationally representative British cohort total energy intake around age 36 years was significantly positively associated with PDA and ADT at age of 51 years in pre- and postmenopausal women [13]. Sala at al. [29] similarly found that total energy intake was significantly positively associated with PDA. The odds ratio (OR) for being classified in the highest PDA category for women in the highest versus lowest tertile of energy intake was 1.79 (95% CI: 1.09–2.91). In analysis stratified by menopausal status, energy intake was associated with significantly higher PDA in postmenopausal women only [29]. Finally, in the Dutch famine study described above, caloric restriction in adulthood was not associated with several BD measures suggesting that exposure to short-term caloric restriction may be more important in children. 

### 5.2. Dietary Fat

Eight studies [24, 25, 27, 29–32, 51] have examined the association between dietary fat and BD in adulthood. Three studies showed a significant positive association with total fat and BD measures. Nagata and colleagues [31] showed significant associations in a Japanese sample with mean PDA being 15.5% in the highest quartile of total fat intake compared to 9.9% in the lowest quartile (*P*_trend_ = 0.04). In a sample of 31 BC patients, women in the highest quartile of total fat (mean % energy (*E*) = 42.04) compared to the lowest quartile of intake (mean %*E* = 34.72) were significantly more likely to be classified as a P2 + DY (high density) pattern compared to the N1 + D1 (low density) pattern (*P* < 0.01) [25]. Qureshi et al. [32] showed a positive trend for the relationship between total fat with increased ADT in a large Norwegian population of postmenopausal women although it did not reach statistical significance.

Individual fatty acids have also been examined with saturated fatty acids (SFAs) generally being positively associated with increased BD measures. In an analysis based on 645 pre- and postmenopausal women ages of 40–62 years enrolled in the Canadian National Breast Screening Study (CNBSS), SFA intake was significantly positively associated with PDA. Mean PDA was 44.2% in the highest quartile of SFA intake compared to 38.6% in the lowest (*P* trend = 0.009) [30]; however, menopausal status was not controlled for or stratified by in this analysis. Similar findings also were reported in pre- and postmenopausal Japanese women; mean PDA was 16.5% in the highest quartile of SFA intake compared to 7.3% in the lowest (*P* trend = 0.02) [31]. Qureshi and colleagues [32] also showed a positive trend with SFA and PDA in a Norwegian population of postmenopausal women although statistical significance was not reached. Nordevang and colleagues [25] observed that women who consumed a mean %*E* of 19.27 from SFA in the highest quartile were more likely to be classified as having a high-risk PDA compared to those who consumed a mean %*E* of 15.42 from SFA in the lowest quartile (*P* ≤ 0.05). In contrast, a significant inverse association was observed with SFA in a subset of 283 premenopausal women from the MBCFSC; mean PDA was 37% in those with the highest SFA intake compared to 44% in the lowest consumers after controlling for relevant confounders (*P* trend = 0.03) [51]. No associations with dietary fat were observed in postmenopausal women alone in this study.

 The essential PUFA, linolenic acid, was inversely associated with PDA in a mediterranean population of both pre- and postmenopausal women. Women in the highest tertile of intake had 31% lower odds of being classified as high PDA [24]. Elevated PUFA consumption in a sample of BC patients (mean %*E* = 5.65 versus 4.70) and n-6 fatty acids (Mean %*E* = 4.69 versus 3.81) was also significantly associated with being classified as a P2 or DY (high density) versus an N1 or P1 (low density) Wolfe parenchymal pattern (*P* < 0.05). Vachon et al. [51] examined a sample of both pre- and postmenopausal women in the MBCFSC and observed women in the highest quartile of PUFA intake had 4% higher PDA compared to those in the lowest quartile (*P* = 0.05). Similar results were observed with the PUFA : SFA ratio in this study. 

 Finally, Nordevang and colleagues [25] found that women within the highest quartile of MUFA (mean %*E* = 14.22) were more likely to have a high PDA compared to those in the lowest quartile (mean %*E* = 11.98, *P* < 0.01). Far fewer associations between dietary factors and BD measures were observed in postmenopausal women, with only increased consumption of MUFAs being significantly associated with high PDA even though the difference in MUFAs as percent energy between the high and low density groups was small (mean %*E* = 12.9 versus 12.3, *P* < 0.05). A small number of randomized controlled trials (RCTs) have also been conducted to examine dietary fat and BD and have yielded mixed results [35–37]. These studies will be further discussed in the “RCT” section of this paper. 

### 5.3. Alcohol

In their 2001 review, Singletary and Gapstur [52] concluded that there was strong evidence for a positive association between alcohol and BD in both pre- and postmenopausal women [52]. Alcohol may influence BD through decreasing the concentration of sex-hormone binding globulin and disturbing estrogen metabolism, increasing serum estrogen metabolites, raising oxidative stress in tissue, and leading to an increase in breast tissue proliferation [53]. The relationship between alcohol and BD may also be related to its positive association with IGF-1 and a negative association with IGFBP-1 that has been shown in post-, but not premenopausal, women [54]. Total alcohol consumption in a multiethnic cohort was associated with a 1-2% higher PDA among pre- and postmenopausal alcohol consumers (median alcohol consumption in the highest consumers = 12 drinks/wk) when compared to abstainers; however, this association failed to reach statistical significance [28]. In a mediterranean cohort of both pre- and postmenopausal women, both total wine consumption and total alcohol consumption were significantly positively associated with a 31% and 42% higher odds of having an elevated PDA, respectively [24]. A similar observation was made with total alcohol consumption in premenopausal women with “Never Drinkers” having a mean PDA of 39% compared 45% for consumers of ≤3.9 g/d and 42% for consumers of >3.9 g/d (*P*_trend_ = 0.08). When the type of alcohol was examined, comparable results were observed with white wine in postmenopausal women only; however, an inverse association was observed with red wine in postmenopausal women with “nondrinkers” having a mean PDA of 34% compared to 32% for those consuming ≤1 serving/wk and 28% for those consuming ≥2–4 svg/wk (*P*_trend_ = 0.02) [51]. The authors suggest that the difference between white and red wine may be due to the polyphenols that are present in red wine, which have been shown to have chemoprotective effects [51]. Tseng et al. [27] and Sala et al. [29] also looked at alcohol intake in pre- and postmenopausal women and found no associations with BD measures. 

### 5.4. Soy and Isoflavones

Maskarinec et al. [55] conducted a review of the primarily epidemiological evidence on isoflavones and their association with PDA and concluded that soy products have a little-to-no influence on BD measures regardless of the amount of isoflavones they are consuming in the range 0.1–120 mg/d [55]. A meta-analysis of several RCTs that examined that the effect of soy and BD measures was also conducted and will be discussed in the “RCT” section of this paper. 

### 5.5. Calcium and Vitamin D

Vitamin D and calcium have been linked to cellular growth and differentiation in breast tissue [56, 57] and may influence the amount of dense tissue in the breast. Four cross-sectional studies found a significant inverse association between vitamin D and calcium intake, alone or in combination, with BD measures [21–23, 25] in premenopausal women. Nordevang et al. [25] found that lower intakes of calcium (1165 versus 1433 mg/10MJ) were significantly associated with an increased PDA. When examining dietary vitamin D and calcium, Bérubé et al. [22] observed that premenopausal women in the highest categories of both vitamin D (≥100 IU/d) and calcium (≥750 mg/d) intake had 72% lower odds of having high PDA. When intake from both diet and supplements was considered, simultaneous increases of 400 IU of vitamin D/d and 1000 mg of calcium/d were associated with an 8.5% (95% CI: 1.8–15.1%) decrease in PDA in premenopausal women [21]. The association in postmenopausal women was considerably weaker [22] or null [21]. Diorio et al. [23] found comparable results; as dietary vitamin D and calcium increased by 100 IU/d and 250 mg/d, respectively, PDA decreased by 1.8% (*P* < 0.01). Similar results were found when intake from food and supplements were analyzed together. 

Out of the remaining seven studies, two included only postmenopausal women and neither found an association between vitamin D and calcium intake and BD [19, 20]. An additional four studies reported significant associations between vitamin D and calcium overall; however, the results in postmenopausal women were considerably weaker than observed in premenopausal women [14, 26, 27]. Masala et al. [24] observed that Mediterranean women with a higher calcium intake had 33% lower odds of having a high-risk mammographic pattern. No association was observed with vitamin D; however, vitamin D intake in this population was very low [24]. In a nationally representative British cohort, an inverse association between calcium intake and PDA, which were both measured among women in their 50's, was observed. Calcium intakes ≥1180 mg/d compared to 699 mg/d resulted in a 0.53 (95% CI: 0.03–1.02) standard deviation decrease in PDA [14]. No additional associations were observed with the ADT or ADNT in this study. Tseng and colleagues [27] conducted a cross-sectional analysis using a 126-item FFQ to examine several dietary factors including vitamin D and found that, after controlling for menopausal status, high-risk women (women with at least one 1st or 2nd degree relative with breast or ovarian cancer) with higher vitamin D intake had 50% lower odds of having high PDA when comparing the highest to the lowest tertile. Finally, serum 25[OH]D and dietary calcium intake obtained from an FFQ in a sample of women from the MBCFSC (73% postmenopausal) were not associated with either PDA or ADT [26]. While the overall trend failed to reach significance, the study did demonstrate that women with the highest mean intake of both calcium (>1,385 mg) and 25(OH)D (>86.2 nmol/L) had the lowest PDA and ADT after adjusting for age, BMI, parity, age at first birth, and physical activity. Vachon et al. [51] also reported no associations for calcium and vitamin D from both dietary and supplemental sources with PDA in this cohort. 

Overall, this research suggests that vitamin D and calcium are inversely associated with BD in premenopausal women. It is critical to note that as calcium and vitamin D increased from <500 mg/d and <100 IU/d to >1,750 mg/d and >700 IU/d, respectively, PDA decreased in a dose-response fashion with clinically relevant decreases in PDA between 8 and 12% among premenopausal women [21, 23]. This is comparable to the effect of selective estrogen receptor modulators such as tamoxifen [58]. Importantly, Brisson et al. [59] examined serum vitamin D [25(OH)D] levels and found that PDA was lowest in the fall (39%) and highest in the spring (45%) (*P* = 0.003), which was consistent with the rise and fall in serum vitamin D across the seasons. Few studies account for season in which BD was assessed. However, it may be important to consider endogenous vitamin D synthesis in response to sunlight in addition to that contributed by food sources. The biologically active form of vitamin D may decrease BD via its antiproliferative properties or tissue-specific effects due to breast tissue possessing 1-*α*-hydroxylase, which converts inactive 25(OH)D to active 1,25(OH)_2_D [60]. The localized production of 1,25(OH)_2_D helps to regulate cell growth and promote terminal differentiation which promotes cellular resistance from carcinogenic factors [60]. Premenopausal women have higher levels of estrogen, insulin-like growth factor (IGF), and insulin-like growth factor binding proteins (IGFBPs), which may be associated with increased BD [61, 62]. Vitamin D, calcium, and IGFBP-3 have been proposed to increase each other's beneficial antiproliferative and proapoptotic effects [23]; however, vitamin D alone may help to combat the proliferative effects of estrogen and IGF when these hormones and growth factors are available in abundance, such as in premenopausal women.

### 5.6. Carbohydrates, Protein, and Other

Ten studies have evaluated intakes of carbohydrates, protein, and many other nutrients and their association with BD measures. Eight studies [16, 24, 27, 30–32, 34, 51] used validated FFQs to assess nutrient intake; Sala et al. [29] and Nordevang et al. [25] conducted extensive dietary history interviews. Tseng and colleagues [27] found that, in a sample of 90 women with a sporadic family history of BC, total and animal protein intakes above the median intake had from 3 to 4 times the odds of an increased PDA; these associations were not observed in women with a strong hereditary pattern (1st or 2nd degree relative) of BC [27]. As mentioned previously, red meat intake during adolescence was significantly positively associated with PDA in adulthood; however, there was no association with red meat intake during adulthood in a sample of 201 Chinese-American immigrants [16]. 

Although few significant associations are observed among postmenopausal women; both Nagata et al. [31] and Sala et al. [29] found significant associations in both Japanese and European populations, respectively, when evaluating carbohydrates and protein. Sala and colleagues [29] found that protein and carbohydrate were positively associated with PDA in all women. When, stratifying by menopausal status, significant positive associations emerged between protein, total meat, and carbohydrates and PDA in postmenopausal women only with those consuming the most having 2.2–2.5 times the odds of having a high-risk PDA. Nagata and colleagues [31] also found that protein was significantly positively associated with PDA with women in the highest quartile of intake having approximately 7% higher PDA than those in the lowest quartile. However, in contrast to the study by Sala, carbohydrates were significantly inversely associated with PDA in 253 postmenopausal Japanese women with those in the highest quartile having 6% lower PDA than the lowest consumers [31]. No associations were observed in premenopausal women [31]. Among pre- and postmenopausal women in the CNBSS, mean PDA was 37.9% in those in the highest quartiles of fiber intake compared to 43.0% in the lowest quartile, and the difference was significant [30]. Comparable results were found in a sample of 31 Swedish premenopausal BC patients; lower consumption of carbohydrate and fiber was associated with higher PDA [25]. 

In a study evaluating dietary factors and mammographic patterns in a Mediterranean population, both pre- and postmenopausal women in the highest tertiles of the following foods and nutrients had 27–34% lower odds of having a high PDA: total vegetables, cheese, *β*-carotene, vitamin C, and potassium, whereas women in the highest tertile of tomato sauce intake had 34% higher odds of having a high PDA [24]. Similar results with high cheese intake were observed in a sample of 491 premenopausal women in this study [24]. Consistent with these findings, total dairy intake was significantly inversely associated with PDA in premenopausal women in the MBCFSC after controlling for relevant confounders [51]. Among pre- and postmenopausal women in the CNBSS, women in the highest quartiles of carotenoid intake had a 5.4% lower mean PDA when compared to the lowest quartile [30]. Comparable results were found by in sample of 31 Swedish premenopausal BC patients and found that lower consumption of carotene was associated with increased PDA [25]. 

Only one study to date has examined multivitamin/multimineral (MVMM) supplement intake and BD outcomes. Bérubé and colleagues [34] found that current premenopausal supplement users had a significantly higher adjusted mean PDA of 45% compared to 42.9% of past or 40.2% of never users (*P*_trend_ = 0.009). No association was observed in postmenopausal women. Vachon et al. [51] also found that dietary vitamin E and supplemental vitamin C were significantly positively associated with PDA in premenopausal women with the highest consumers having a 4-5% higher PDA than the lowest consumers. Supplemental vitamin B12, on the other hand, was positively related to PDA in postmenopausal women [51]. 

In conclusion, the foods or nutrients that were shown to be inversely associated with BD may be, in part, tied to IGF/IGFBP levels and oxidative stress reduction. BD has been associated with increased levels of oxidative stress as evidenced by malonoyldialdhyde (MDA) excretion [63] and IGF/IGFBP, particularly in premenopausal women [50, 61]. Lower intakes of fiber, carotene, and calcium have also been associated with increased breast densities. Carbohydrate intake has been associated with both lower and higher BD measures women. These conflicting results may be attributed to the fact that the types of carbohydrate are often not accounted for and fiber content may influence the way that different carbohydrates affect the IGF/IGFBP pathway and oxidative stress. Finally, higher intakes of total dairy and cheese consumption in premenopausal women are associated with lower BD measures, which may be due to the high amounts of calcium and vitamin D in these products. 

### 5.7. Dietary Patterns

 Analysis of dietary patterns has recently gained popularity in dietary assessment research, as they capture total diet and are more stable over time than the consumption of single nutrients or foods [64]. Two studies were conducted that examined *a posteriori* dietary patterns and their association with BD and one study examined the influence of Mediterranean Diet (measured by Mediterranean diet scale (MDS)) on BD measures. Dietary patterns were analyzed cross-sectionally in a British cohort and the MBCFSC [13, 33]. After combining data collected from food records collected at ages 36 and 43 years, four patterns emerged in the British cohort ((1) low-fat and high fiber; (2) alcohol and fish; (3) high fat and sugar; (4) meat, potatoes, and vegetables). However, none of these patterns was associated with PDA [13]. In the MBCFSC, three dietary patterns emerged from data from a 153-item FFQ ((1) fruit, vegetable, and cereal; (2) salad, sauce, and pasta/grain; (3) meat and starch). Only the fruit, vegetable, and cereal pattern was inversely associated with PDA in premenopausal women; however, it did not reach statistical significance [33]. Smoking has been associated with decreased PDA because of its antiestrogenic effects [65]. When all women included in the sample were stratified by smoking status, adherence to the fruit, vegetable, and cereal pattern was significantly inversely associated with PDA in smokers (*P* = 0.02) [33]. The salad, sauce, and pasta/grain pattern was also nonsignificantly inversely associated with PDA in smokers [16]. These patterns are the highest in antioxidant-containing foods, which may benefit women who are under higher oxidative stress, such as smokers. 

Tseng et al. [66] cross-sectionally evaluated the MBCFSC using the MDS. The women were scored based on their consumption of vegetables, legumes, fruits and nuts, cereals, fish, and the ratio of monounsaturated fatty acids (MUFA) to saturated fatty acids (SFA) as reported on a 153-item FFQ. For each unit increase in the MDS, PDA was decreased by 1.68% (*P* = 0.0002) among current smokers but not among nonsmokers after controlling for relevant confounders including menopausal status [66]. Vegetables, legumes, and cereals were the components of the MDS that had the strongest association with PDA in this population [66].

Overall, it appears that dietary patterns high in antioxidant-containing foods are inversely associated with BD in smokers, who may be experiencing a higher level of oxidative stress than nonsmokers. Other research has shown a positive association between BD and MDA, which is a marker for lipid peroxidation and oxidative stress [63]. 

### 5.8. Randomized Controlled Trials

The epidemiological evidence described above suggests that diet is associated with BD measures and that BD has the potential to be modified. As a result, researchers have conducted clinical trials to examine the association between specific dietary factors with BD outcomes (Table 8). Boyd et al. [35] first examined a low-fat, high-carbohydrate 2-year dietary intervention in 817 women with PDAs ≥50%. Those who were randomized into the intervention group received intensive instruction to consume 15% of calories from fat, 20% from protein, and 65% from carbohydrate while the control group received general dietary advice and instruction to maintain their current intake of fat. After two years, the average reduction in PDA was 6.1% and 2.1% in the intervention and control groups, respectively, (*P* = 0.01) [35]. The effect of the intervention remained significant after controlling for age, weight change, and menopausal status [35]. After stratification by menopausal status, significant changes in PDA were only observed in women who were either premenopausal throughout the study or who were premenopausal at baseline but transitioned into menopause by the end of the study, with the greatest change in density occurring in the latter group. Consumption of fat and cholesterol was significantly positively associated with change in ADT in this subgroup, whereas protein and cholesterol were significantly positively associated with change in PDA [37]. 

Martin et al. [36] completed a similar larger clinical trial with longer followup that included 461 women who were premenopausal at entry and postmenopausal after two years. Several BD measures were assessed (change in breast area, ANDT, ADT, PDA) premenopausally at baseline and later in the postmenopausal phase. Like the previous trial, this trial focused on women with high PDA ≥50% and the intervention group received the same dietary manipulation [35]. This study did not replicate the previous findings from Boyd et al. [35]. After two years, no change was observed in the intervention group and a slightly lower PDA was observed in the control group; the treatment group difference was not significant [36]. The authors suggest that these unexpected results were likely due to an increase in the ANDT that occurred with weight gain in the sample. 

As previously described in the vitamin D and calcium section, a one-year calcium and vitamin D supplementation trial was conducted through the WHI to examine the effects on mammographic PDA in postmenopausal women [20]. Despite the associations observed in observational studies, no change in mammographic PDA was observed with supplementation. The authors suggest that very low PDAs at baseline could have led to a “floor effect” where further supplementation of vitamin D and calcium had no additional benefit. Finally, studies that have examined soy and isoflavone consumption and mammographic PDA have also yielded mixed results. Hooper et al. [67] conducted a meta-analysis of eight RCTs including 1287 total women that compared the administration of supplemental isoflavones versus a placebo for at least six months. Results from the meta-analysis showed a modest nonsignificant increase in PDA (mean difference: 1.83%; 95% CI 0.25–3.40) in premenopausal, but not postmenopausal, women as isoflavone intake increased; however, there was limited evidence of a clear dose-response relationship over the range of isoflavone intake of 40–120 mg/d. 

## 6. Conclusions

 Data from observational studies suggest that the strongest associations between diet and BD measures are among vitamin D, calcium, dietary fat, and alcohol and are found in adult premenopausal women. However, the few clinical trials that have evaluated these associations have failed to demonstrate a significant change in breast density with various dietary interventions. This could be because the foods/nutrients evaluated truly do not influence breast density or could be due to aspects of the study design including duration of the intervention, dose, sample size, or inclusion of predominantly older women in whom breast tissue may be less susceptible to dietary influences. 

### 6.1. Limitations

This paper has critically examined 28 studies and has identified strengths and weaknesses as well as highlighting several potential directions for new research to advance the field. Many of these studies are cross-sectional in nature and often focus just on PDA. In addition to this, the majority of women who receive mammograms overall and in these studies are >40 y; an association between dietary factors and BD measures could be undetected if the critical dietary exposure occurred much earlier in life (and was not measured) before breast tissue is fully differentiated and potentially more vulnerable to exogenous influences.

The majority of studies included in the paper assessed BD using 2D mammography. Even though estimates of BD obtained by mammography and 3D modalities such as MRI are highly correlated in the general population and in women with less dense breasts [38], correlations are substantially lower in women with more dense breasts in whom density can be more accurately measured using 3D modalities. 

 Many studies examined the association of diet with PDA but not the ADT. Fewer associations are observed with the ADT compared to PDA; however, results should be reported when available in order to be more comprehensive, improve comparisons across studies, and enhance interpretability in relation to potential physiological mechanisms. Very few studies controlled for the phase of the menstrual cycle at the time of mammography. Because data on variation of breast density over the menstrual cycle are conflicting [68–71], it seems prudent to consider menstrual cycle day in analyses of breast density when possible. Finally, several methods were used to evaluate BD. Even though many studies used a semiautomated method to reduce variability and error, standardization of assessment would facilitate comparisons across studies. 

### 6.2. Future Directions

To date, most studies of the association of diet with BD have been cross-sectional. Longitudinal studies that measure diet and BD over the life course are needed. Studies that evaluate the influence of diet during adolescence, when most breast development occurs, on adult BD could be particularly enlightening. Support for an association of diet with BD from observational studies is stronger for premenopausal women. However, a limited number of short-term clinical trials do not show conclusive evidence that dietary factors influence BD. Clinical trials in younger women could be informative and may provide more definitive results. Lastly, more research on dietary patterns as they relate to BD are needed. 

## Figures and Tables

**Table 1:** Studies of childhood diet and breast density.

Author, year	Study population, *n*	Design	Diet/mammogram Age	Foods/nutrients of interest	Dietary assessment	Outcome	Major significant results	Adjustments
Haars, et al. 2010 [12] (also in Tables 2 and 9)	DOM-Project *n* = 144 (The Netherlands)	CS	2–9 y/53 y	Short-term energy restriction	Retrospective recall of 1944-45 famine ~ 40 yrs later. Exposure to hunger, cold, and weight loss.	BS, DT, NDT, PBD (mammogram; visual observation)	Severely calorically restricted versus unrestricted: NDT: 53.1 cm^2^ (95% CI: 37.8–72.7) versus 77 cm^2^(95% CI: 68.8–87.7)	Age at examination, parity, menopausal status, BMI

Mishra, et al. 2011 [13] (also in Tables 9 and 6)	BBC *n* = 792 (England)	PC	4 y/51.5 y	Dietary patterns at age 4: (1) breads and fats (2) fried potatoes and fish, (3) milk, fruit, and biscuits	1–24-hr maternal recall of child's diet	PBD, ADT, ANDT (mammogram; Cumulus)	Null	Mammographic view, age at mammogram, BMI at 53, age at menarche, menopausal status at mammography, HT use, parity, smoking status, PA, social class, the other three dietary patterns, energy

Mishra, et al. 2008 [14] (also in Table 3)	BBC *n* = 979 (England)	PC	4 y/51.5 y	Dietary Ca and vitamin D	1–24-hour maternal recall of child's diet	PBD, ADT, ANDT (mammogram; Cumulus)	Null	Mammographic view, age at mammogram, BMI age 53, energy, age at menarche, parity, smoking status, adult SES.

**Table 2:** Studies of adolescent diet, and breast density.

Author, year	Study population, *n*	Design	Diet/mammogram age	Foods/nutrients of interest	Dietary assessment	Outcome	Major significant results	Adjustments
Haars et al. 2010 [12] (also in Tables 1 and 9)	DOM-project *n* = 356 (The Netherlands)	CS	10–18 y/53 y	Short-term energy restriction	Described in Table 1	BS, DT, NDT, PBD (mammogram; visual observation)	Null	Age at examination, parity, menopausal status, BMI

Sellers et al. 2007 [15]	MBCFSC *n* = 1,552 (United States, NH-White)	CS	12-13 y/60.4 y	High-fat meats, dairy, animal fat, high-fat snacks and desserts, high-fat foods, fish and chicken, fruits, vegetables	29-item FFQ (retrospective recall)	PBD (mammogram; Cumulus)	Null	Age at mammography, weight at follow-up 1, use of HRT, menopausal status, education, age at menarche, parity, age at first birth, OC use, alcohol use, smoking hx

Tseng et al. 2011 [16] (also in Table 7)	Chinese-American immigrants *n* = 201 (US, Asian)	CS	12–17 y/53.1 y	Beef, pork, tofu, green veg, fruits	Frequency of consumption: beef, pork, tofu, green veg, fruits (retrospective)	PBD: BIRADS	Red meat intake: all women: (OR_*T3* versus *T1*_ = 3.0; 95% CI: 1.5–6.4) postmenopausal women: (OR_*T3* versus *T1*_ = 16.9; 95% CI 5.4–52.4)	Age, level of acculturation, BMI, number of live births and age at first live birth, adult dairy intake

Vachon et al. 2005 [17]	MBCFSC *n* = 1575 (US, NH-White)	CS	<18 y/60.4 y	Alcohol	Follow-up questionnaire	PBD (mammogram; Cumulus)	Null	Age, BMI, HRT, age at first birth, number of births, age at menarche, education, adult and adolescent smoking status, alcohol, OC use, menopausal status

Dorgan et al. 2010 [18] (also in Table 8)	DISC premenopausal women *n* = 182 (US, NH-White)	CS (RCT followup)	25–29 y	Long-term effects of low-fat diet	3–24-hr dietary recalls	PBD and VDT (MRI)	Null	% body fat, age at randomization, age at visit, clinic, BMI-*Z* score, race, education, smoking status, PA at 14–17 years old and separately during the past year, number of full-term pregnancies, hormonal contraceptives

**Table 3:** Studies of adult calcium and vitamin D intake and breast density.

Author, year	Study population, *n*	Design	Age	Foods/nutrients of interest	Dietary assessment	Outcome	Major significant results	Adjustments
Bertone-Johnson, et al. 2010 [19]	MDAS: WHI *n* = 808 Postmenopausal (US, 42% NH-White, 39% Black, 20% other races)	CS	50–79 y	Dietary and supplemental vitamin D and Ca	122-item FFQ + supplement inventory	PBD: (mammogram; computer-assisted method)	Null	Age, race/ethnicity, BMI, age at menarche, parity, OC use and duration, previous HT use/duration, HT trial randomization assignment, family hx of BC, education, alcohol, smoking, total energy, PA, Gail risk, MV use

Bertone-Johnson et al. 2012 [20] (also in Table 8)	WHI CaD trial *n* = 330 postmenopausal women (US, 48% NH-White, 36% Black, 15% other)	RCT	50–79 y	Daily supplementation of both 1,000 mg of Ca and 400 IU of vitamin D (1y)	122-item FFQ	PBD: (Mammogram: computer-assisted method)	Null	Subgroup analyses: age, race/ethnicity, total vitamin D intake, HT treatment, Gail risk score, BMI, region of residence, category of mammogram density at baseline.

Bérubé et al. 2005 [21]	Premenopausal women: *n* = 777 Postmenopausal: *n* = 783 (Canada)	CS	Premenopausal: 46.7 y Postmenopausal: 61.8 y	Dietary and supplemental vitamin D and Ca	161-item FFQ	PBD: (mammogram; computer-assisted method)	Premenopausal women: dietary vitamin D: *β* = −1.8; total vitamin D: *β* = −1.4; dietary calcium: *β* = −0.7; total calcium: *β* = −0.8. 8.5% ↓mean PBD with simultaneous increases in VD and Ca by 400 IU and 1,000 mg, respectively. Postmenopausal women: null All women: absolute ↓ in mean PBD_*Q*4Ca and VitDQ1Ca and VitD_= 6.9%,	Age, BMI, age at menarche, number of full-term pregnancies, age at first full-term pregnancy, duration of OC and/or HRT use, alcohol, daily energy, PA, family hx of BC in 1st degree relative, personal history of breast biopsies, smoking status, education (supplement use was also a confounder, determined post hoc)

Bérubé et al. 2004 [22]	Pre- and postmenopausal women with extreme densities *n* = 543 (US)	CS	PBD ≤ 30%: 51.4 y PBD ≥ 70%: 46.1 y	Vitamin D and dietary Ca	232-item FFQ	PBD: (mammogram; visual estimation)	All women: vitamin D: OR_*Q*4 versus *Q*1_= 0.24 (95% CI: 0.11–0.53); calcium: OR_*Q*4 versus *Q*1_ = 0.24 (95% CI: 0.10–0.57) OR_EXT versus FEW DENSITIES_ 0.28 (95% CI: 0.15–0.54) (≥100 IU Vit D and ≥750 mg/d Ca)=)premenopausal women: vitamin D:OR_*Q*4 versus *Q*1_ = 0.13; calcium: OR_*Q*4 versus *Q*1_ = 0.13 postmenopausal women: vitamin D: OR_*Q*4 versus *Q*1_ = 0.30 (*P* trend = 0.05) calcium: OR_*Q*4 versus *Q*1_ = 0.27 (*P*-trend = 0.06)	Age, mammography, BMI, age at menarche, number of births and age at first birth combined, OCs, menopausal status and use of HRT combined, family hx of BC, education, alcohol, total energy, smoking status

Diorio et al. 2006 [23]	Premenopausal women *n* = 771 (Canada)	CS	<48 y (if a nonsmoker) and <46 y (if a smoker)	Dietary and supplemental vitamin D and Ca	FFQ	PBD: (mammogram: computer-assisted method)	Food only: vitamin D: *β* for 100 IU/d = −1.8; calcium: *β* for 250 mg/d = −1.8 Food and supp: vitamin D: *β* for 100 IU/d = −1.4; calcium: *β* for 250 mg/d = −1.9	Alcohol, total energy, age, BMI, age at menarche, age at first full-term pregnancy, number of full-term pregnancies, number of breast biopsies, duration of past use of OC and of HRT, family history of BC in 1st degree relative, PA, education, smoking status

Masala et al. 2006 [24] (also in Tables 4, 5, and 7)	Mediterranean population—florence section of EPIC *n* = 1, 668 (Italy)	CS	Pre-, post-, and perimenopausal women	Vitamin D and Ca	160-item validated FFQ	Wolfe classification (P2 + DY versus N1 + P1) and semiquantitative method	All women: P2 + DY versus N1 + P1: calcium OR_*T*3 versus *T*1_ = 0.67 (95% CI: 0.47–0.94)	Age, education, BMI, menopausal status, total energy (log), each food separately (tertiles)

Mishra et al. 2008 [14] (also in Table 1)	BBC (*n*'s ranged from 674 to 979 women) *Cross-sectional n:*total: *n* = 287 (England)	PC	36, 43, 53 y/51.5 y	Dietary Ca and vitamin D (age 53 follow-up: included supplement data)	5-day food records	PBD, ADT, ANDT (mammogram; cumulus)	Null *cross-sectional findings:* postmenopausal women: ≥1180 mg/d^−1^ versus ≤699 mg/d^−1^, 0.53 s.d. lower PBD (95% CI: 0.03–1.02)	Mammographic view, age at mammogram, BMI at 53, energy, age at menarche, parity, smoking status, adult SES

Nordevang et al. 1993 [25] (also in Tables 5 and 7)	BC patients (stage I-II) *n* = 238 (Sweden)	CS	57.5 y	Ca	Dietary hx interview within 4 months of BC diagnosis	Wolfe classification (N1 + P1 versus P2 + Dy)	Premenopausal women: P2 + Dy versus N1 + P1: calcium (1165 versus 1433 mg/10 MJ)	BMI, age, ER status

Knight et al., 2006 [26]	MBCFCS *n* = 487 (US, NH-white)	CS	56.4 y	Vitamin D (25(OH)D) and dietary Ca	FFQ	PBD, TDA (mammogram: Cumulus)	Null	Full model: age, BMI, parity, age at first birth, PA

Tseng et al. 2007 [27] (also in Tables 4, 5, and 7)	Women with at least one 1st degree or 2nd degree relative with BC or ovarian cancer *n* = 157 (US, NH-White)	CS	50 y	Vitamin D and Ca	126-item FFQ	PBD: BIRADS	OR: vitamin D intake_*T*3 versus *T*1_, 0.5 (95% CI: 0.2–1.1)	Age, BMI, caloric intake, age at menarche, menopausal status, history of HRT, family history category.

Vachon et al., 2000 [9] (also in Tables 4, 5, and 7)	MBCFCS *n* = 1508 (US, NH-White)	CS	61.4 y	Vitamin D and Ca	153-item validated FFQ	PBD (Mammogram: visual estimation)	null	Energy, age, BMI, WHR, PA, age at menarche, age at first birth and number of births (combined), alcohol, smoking, family hx of BC, HRT (all and postmenopausal women) and OC use (premenopausal women)

**Table 4:** Studies of alcohol intake in adulthood and breast density.

Author, year	Study population, *n*	Design	Age	Foods/nutrients of interest	Dietary assessment	Outcome	Major significant results	Adjustments
Maskarinec et al. 2006 [28]	BEAN (*n* = 217 premenopausal women) and MEC (*n* = 582 cases and *n* = 658 controls) (multiethnic cohort)	BEAN = CS MEC = CC	BEAN = 43 y MEC (cases and controls) = 57 y	Alcohol	Validated FFQ	Mammogram: (computer-assisted method)	Null	Age, BMI, ethnicity, HRT use, age at first live birth, parity, age at menarche, menopausal status, group status, family hx of BC when appropriate.

Masala et al. 2006 [24] (also in Tables 3, 5, and 7)	Mediterranean Population—florence section of EPIC *n* = 1,668 (Italy)	CS	Pre-, post-, and peri-menopausal women	Alcohol	160-item validated FFQ	Wolfe classification (P2 + DY versus N1 + P1) and semiquantitative method (“entirely fat”; <25%, “25–75%, >75% high density area)	All Women: P2 + DY versus N1 + P1: Overall alcohol: OR_*T*3 versus *T*1_ = 1.31 (95% CI: 1.01–1.72) Premenopausal women at enrollment (*n* = 491): Wine: OR_*Q*4 versus *Q*1_: 1.84 (95% CI: 1.07–3.16); high alcohol consumption OR_*Q*4 versus *Q*1_: 1.86 (95% CI: 1.03–3.38)	Age, education, BMI, menopausal status, total energy (log), each food separately (tertiles)

Sala et al. 2000 [29] (also in Tables 5 and 7)	EPIC-Norfolk Cases: P2/DY Controls: N1/P1 (*n* = 203 cases and *n* = 203 controls) (UK)	NCC	Cases and controls: 59 y	Alcohol	7-day food record	Wolfe Patterns: (high risk: P2 and DY; low risk: N1 and P1)	Null	Menopausal status, parity, HRT, BMI

Tseng et al., 2007 [27] (also in Tables 3, 5, and 7)	Women with at least one 1st degree or 2nd degree relative with BC or ovarian cancer *n* = 157 (US, NH-White)	CS	50 y	Alcohol	126 item validated FFQ	PBD: BIRADS	Null	Age, BMI, energy, age at menarche, menopausal status, hx of HRT, family hx category.

Vachon et al. 2000 [9] (also in Tables 3, 5, and 7)	MBCFCS *n* = 1508 (US, NH-White)	CS	61.4 y	Alcohol	153-item validated FFQ	PBD (Mammogram: visual estimation)	Postmenopausal women: white wine: nondrinkers versus ≥2–4 svg/wk = 29% (95% CI: 26–32%) versus 34% (95% CI: 30–37%), Red wine: nondrinkers versus ≥2–4 svg/wk: 34% (95% CI: 31–36%) versus 28% (95% CI: 24–33%)	Energy intake, age, BMI, WHR, PA, age at menarche, age at first birth and number of births (combined), alcohol, smoking, family hx of BC, HRT (all and postmenopausal women), OC (premenopausal women)

**Table 5:** Studies of dietary fat intake in adulthood and breast density.

Author, year	Study population, *n*	Design	Age	Foods/Nutrients of interest	Dietary assessment	Outcome	Major significant results	Adjustments
Brisson et al. 1989 [30] (Also in Table 7)	CNBSS—newly Diagnosed BC patients cases: *n* = 290 controls: *n* = 645 total *n* = 935 (Canada)	CC	40–62 y	Dietary fats	114-item FFQ + questions on vitamin A	Wolfe classification (high risk: P2 + DY; low risk: N1 + P1) (mammogram: visual estimation)	Controls (total densities): saturated fat_*Q*4 versus *Q*1_: 44.2% versus 38.6%, *β* = 0.370 (SE = 0.141)	Age, body weight, parity, education, energy

Masala et al. 2006 [24] (also in Tables 3, 5, and 7)	Mediterranean population—florence section of EPIC *n* = 1,668 (Italy)	CS	Pre-, post-, and perimenopausal women	Dietary fats	160-item FFQ	Wolfe classification (P2 + DY versus N1 + P1) & semiquantitative method	All women: P2 + DY versus N1 + P1: Olive Oil OR_*T*3 versus *T*1_ 0.73 (95% CI: 0.55–0.98) linolenic acid OR_*T*3 versus *T*1_ = 0.69 (95% CI: 0.47–0.99, *P* trend = 0.05)	Age, education, BMI, menopausal status, total energy (log), each food separately (tertiles)

Nagata et al. 2005 [31] (also in Table 7)	Japanese women *n* = 601 (Japan)	CS	Premenopausal women: 42.6 y Postmenopausal women: 57.8 y	Dietary fats	169-item FFQ	PBD (Mammogram: fully-automated method)	Postmenopausal women: Total Fat: *Q*4 versus *Q*1 = 15.5 (95% CI: 10.8–21.2) versus 9.9% (95% CI: 6.8–13.7; Saturated fat: *Q*4 versus *Q*1 = 16.5% (95 CI:11.3–22.6%) versus 7.3% (95% CI: 4.7–10.4%)	Age, BMI, smoking status, number of births, hx of breast feeding for premenopausal women and for age, BMI, education, age at menopause for postmenopausal women. Nutrient intakes were adjusted for total energy.

Nordevang et al. 1993 [25] (also in Tables 3 and 7)	BC Patients (stage I-II) *n* = 238 (Sweden)	CS	57.5 y	Dietary fats	Dietary history interview within 4 months of BC diagnosis	Wolfe classification (N1 + P1 versus P2 + Dy)	Premenopausal women: P2 + Dy versus N1 + P1: total fat (42.04 versus 34.72% *E*); saturated fat (19.27 versus 15.42% *E*), MUFA (14.22 versus 11.98% *E*); PUFA (5.65 versus 4.70), n-6 FA (4.69 versus 3.81% *E*) postmenopausal women: P2 + Dy versus N1 + P1: MUFA (12.88 versus 12.32% *E*)	BMI, age, ER status

Sala et al. 2000 [29] (also in Tables 4 and 7)	EPIC-Norfolk Cases: P2/DY Controls: N1/P1 (*n* = 203 cases and *n* = 203 controls) (UK)	NCC	Cases and controls: 59 y	Dietary fats	7-day food record	Wolfe patterns: (high risk: P2 & DY; low risk: N1 & P1)	Null	Menopausal status, parity, HRT, BMI

Tseng et al. 2007 [27] (also in Table 3, 4, and 7)	1st degree or 2nd degree relative with BC or ovarian cancer *n* = 157 (US, NH-White)	CS	50 y	Dietary fats	126 item validated FFQ	PBD: BIRADS	Null	Age, BMI, caloric intake, age at menarche, menopausal status, history of HRT, family history category.

Qureshi et al. 2011 [32] (also in Table 7)	NBCSP *n* = 2,252 Postmenopausal women (Norway)	CS	58 y	Dietary fats	180-item validated FFQ	PBD & AD (mammogram: computer-assisted method)	PBD: Saturated fat_*Q*4 versus *Q*1_: 19.7 (95% CI: 18.7–20.7%) versus 17.0 (95% CI: 15.6–18.3, *P*-trend = 0.06)	Age at mammography, y of education, age at menarche, number of pregnancies, age at first full-term pregnancy for parous women, HRT, BMI, total energy

Vachon et al. 2000 [9] (also in Tables 3, 4, and 7)	MBCFCS *n* = 1508 (US, NH-White)	CS	61.4 y	Dietary fats	153-item FFQ	PBD (mammogram: visual estimation)	Premenopausal women: PUFAs: *Q*4 versus *Q*1: 42% (95% CI: 35–49%) versus 38% (95% CI: 37–51%) PUFA : SFA: 43% (95% CI: 36–50%) versus 38% (33–44%,); SFA: *Q*4 versus *Q*1: 37% (95% CI: 32–43%) versus 44% (95% CI: 37–51%)	Energy, age, BMI, WHR, PA, age at menarche, age at first birth and number of births (combined), self-reported alcohol intake, smoking, family hx of BC, HRT (all and postmenopausal women), OC (premenopausal women)

**Table 6:** Studies of dietary patterns in adulthood and breast density.

Author, year	Study population, *n*	Design	Age	Foods/nutrients of interest	Dietary assessment	Outcome	Major significant results	Adjustments
Mishra et al. 2011 [13] (also in Table 1)	BBC *n* = 700 (England)	PC	36, 43 y/51 y (“habitual adult” dietary patterns)	Dietary patterns: (1) low fat, fiber (2) alcohol and fish (3) high fat and sugar (4) meat, potatoes, and vegetables	5-day food records	PBD, ADT, ANDT (Mammogram; Cumulus)	Null	Mammographic view, age at mammogram, BMI at 53, age at menarche, menopausal status at the time of mammography, HT use, parity, smoking status, PA, social class, other three dietary patterns, energy

Tseng et al. 2008 [33]	MBCFSC *n* = 1,286 (US, NH-White)	CS	57 y	MDS	153-item validated FFQ	PBD (Mammogram: semiautomated threshold method)	CCurrent smokers (*n* = 176) and the MDS (continuous): *β* = −1.68 (SE = 0.55) MDS category: *β*_CAT3 versus CAT1_ = −7.17 (SE = 2.77)	Age, total energy, menopausal status, education, HRT, BMI, WHR, age at menarche, parity and age at first live birth (combined variable), alcohol, relation to proband

Tseng et al. 2008 [33]	MBCFSC *n* = 1, 286 (US, NH-White)	CS	57 y	Dietary patterns: (1) fruit-vegetable-cereal pattern (2) salad-sauce-pasta/grain pattern (3) meat-starch pattern	153-item validated FFQ	PBD (Mammogram: semiautomated method)	Smokers: fruit-vegetable-cereal pattern: *β* = −0.30 (SE = 0.13) Salad-sauce-pasta/grain pattern: (*β* = −0.27) (SE = 0.15, *P* = 0.06)	Age, total energy, menopausal status, education, PA, HRT, BMI, WHR, age at menarche, parity and age at first birth, alcohol, relation to proband

**Table 7:** Studies of selected nutrients in adulthood and breast density.

Author, year	Study population, (*n*)	Design	Age	Foods/nutrients of interest	Dietary assessment	Outcome	Major significant results	Adjustments
Bérubé et al. 2008 [34]	Premenopausal women: *n* = 777 Postmenopausal women: *n* = 783 (Canada)	CS	Premenopausal: 47 y Postmenopausal: 60 y	MVMM supplements	161-item FFQ	PBD: (Mammogram: computer-assisted method)	Premenopausal women: current users (45%, SE: 1.64%), past (42.9%, SE: 1.28%), never users (40.2% SE: 1.05%)	Age, education, BMI, age at menarche, number of full-term pregnancies, age at first full-term pregnancy, duration of OC and HRT, smoking status, PA, family hx of BC in first degree relative, personal hx of breast biopsy, chronic illness, mean energy, alcohol, vitamin and mineral supplements, following special diet, dietary vitamin D and calcium intake, season of mammography

Brisson et al. 1989 [30] (also in Table 5)	CNBSS—newly diagnosed BC patients Cases: *n* = 290 Controls: *n* = 645 Total *n* = 935 (Canada)	CC	40–62 y	Several dietary factors, especially vitamin A	114-item FFQ + additional questions on vitamin A	Wolfe classification (high risk: P2 + DY; low risk: N1 + P1) (Mammogram: visual estimation)	Controls (Total Densities): Carotenoids_*Q*4 versus *Q*1_: 38.2% versus 43.6%, *β* = −392 (SE = 171); Fiber_*Q*4 versus *Q*1_: 37.9% versus 43.0%, *β* = −1.02 (SE = 0.41)	Age, bodyweight, parity, education, energy

Masala et al. 2006 [24] (also in Tables 3, 4, and 5)	Mediterranean Population—Florence section of EPIC *n* = 1,668 (Italy)	CS	Pre-, post-, and peri-menopausal women	Several dietary factors	160-item validated FFQ	Wolfe classification (P2 + DY versus N1 + P1) and semi-quantitative method	All Women: P2 + DY versus N1 + P1: Vegetables: OR_*T3* versus *T1*_ = 0.66 (95% CI:0.50–0.88); Cheese: OR_*T3* versus *T1*_: 0.73 (95% CI: 0.55–0.99); *β*-carotene OR_*T3* versus *T1*_ = 0.71 (95% CI: 0.53–0.94), Vitamin C OR_*T3* versus *T1*_ = 0.75 (95% CI: 0.56–0.99); Potassium OR_*T3* versus *T1*_ = 0.69 (95% CI: 0.48–1.00, *P*-trend = 0.05), Tomato sauce: OR_*T3* versus *T1*_ = 1.34 (95% CI: 1.01−1.77) Premenopausal women at enrollment (*n* = 491): High consumption of cheese: OR_*Q4* versus *Q1*_ 0.44 (95% CI: 0.23–0.84)	Age, education, BMI, menopausal status, total energy(log), each food separately (tertiles)

Nagata et al. 2005 [31] (also in Table 5)	Japanese women *n* = 601 (Japan, Asian)	CS	Premenopausal women: 42.6 y Postmenopausal women: 57.8 y	Protein, dietary fiber, and soy isoflavones	169-item validated FFQ	PBD (mammogram: fully automated method)	Postmenopausal women: protein: *Q*4 versus *Q*1 = 13.9% (95% CI: 10.4–18.0%) versus 6.7% (95% CI: 3.6–10.7%; CHO: *Q*4 versus Q1 = 9.6% (95% CI: 6.5–13.2) versus 15.6% (95% CI: 11.1–20.9%)	Age, BMI, smoking status, number of births, and hx of breast feeding for premenopausal women and for age, BMI, number of births, education, age at menopause; nutrient intakes were adjusted for total energy.

Nordevang et al. 1993 [25] (also in Tables 3 and 5)	BC patients (stage I-II) *n* = 238 (Sweden)	CS	57.5 y	Various nutrients	Dietary history interview within 4 months of BC diagnosis	Wolfe classification (N1 + P1 versus P2 + Dy)	Premenopausal women: P2 + Dy versus N1 + P1: CHO: (40.41 versus 47.37% *E*); Fiber (19.05 versus 26.09 mg/10 MJ), Carotene (3.80 versus 5.62 mg/MJ)	BMI, age, ER status

Sala et al. 2000 [29] (also in Tables 4, 5 and 9)	EPIC-Norfolk cases: P2/DY controls: N1/P1 (*n* = 203 cases and *n*= 203 controls) (UK)	NCC	Cases and controls: 59 y	Vitamin A, vitamin C, vitamin E, protein, carbohydrate, fiber, vegetables, cereals and breads, fruits, red meat, white meat, total meat, milk, dairy products, fish.	7-day food record	Wolfe patterns: (high risk: P2 and DY; low risk: N1 and P1)	All women: protein: OR_OR *T3* versus *T1*_ = 2.00 (95%CI:1.06–3.77)**; total CHO: OR_OR *T3* versus *T1*_ = 1.93, 95% CI: 1.03–3.59)** Postmenopausal women: Protein: (OR_OR *T3* versus *T1*_-= 2.20, 1.04–4.63, *P* = 0.03)**, Total CHO: (OR_OR *T3* versus *T1*_ = 2.22, 1.02–4.79)**, Total meat intake: (OR_OR *T3* versus *T1*_ = 2.50, 1.09 = 5.69)**	*Unadjusted **Menopausal status, parity, HRT, BMI

Tseng et al. 2007 [27] (also in Table 3, 4, and 5)	At 1st degree or 2nd degree relative with BC or ovarian cancer *n* = 157 (US, NH-White)	CS	50 y	Calories, cholesterol, protein, animal protein, carbs, dietary fiber, carotene, folate, vitamin E, meats, fruits, vegetables, tofu.	126 item FFQ	PBD: BIRADS	Women who do not have hereditary cancer patterns: protein (OR: 3.0 (95% CI: 1.3–6.9)) and animal protein (OR: 4.3 (95% CI: 1.8–10.3)	Age, BMI, energy, age at menarche, menopausal status, hx of HRT, family hx category.

Tseng et al. 2011 [16] (also in Table 2)	Chinese-American immigrant women *n* = 201 (US, Asian)	CS	53.1 y	Red meat	88-item FFQ	PBD: BIRADS	Null	Age, level of acculturation, BMI, combined variable representing # of live births and age at first live birth, adult weekly frequency of dairy food intake

Qureshi et al. 2011 [32] (also in Table 5)	NBCSP *n* = 2,252 Postmenopausal women (Norway)	CS	58 y	Various nutrients and vitamins	180-item FFQ	PBD and AD (mammogram: computer-assisted method)	PBD: Saturated fat_Q4 versus Q1_: 19.7 (95% CI: 18.7–20.7%) versus 17.0 (95% CI: 15.6–18.3, *P*-trend = 0.06)	Age at mammography, y of education, age at menarche, number of pregnancies, age at first full-term pregnancy for parous women, HRT, BMI, total energy

Vachon et al. 2000 [9] (also in Tables 3, 4, and 5)	MBCFCS *n* = 1508 (US, NH-White)	CS	61.4 y	Vitamin A, retinol, carotene, crude and dietary fiber, total carbohydrates, cholesterol, B12, folate, vitamins C, E, total protein, total energy	153-item FFQ	PBD (mammogram: visual estimation)	Premenopausal women: vit E: *Q*4 versus *Q*1: 42% (95% CI: 36–47%) versus 38% (95% CI: 33–46%, *P* trend = 0.05); total dairy intake: *T*3 versus *T*1 = 38% (95% CI = 32–44%) versus 44% (95% CI: 37–51%) Postmenopausal women: Vit B12 (sup only): *Q*4 versus *Q*1: 34% (95% CI: 31–36%) versus 32% (95% CI: 30–34%, *P* trend = 0.05)	Energy intake, age, BMI, WHR, PA, age at menarche, age at first birth and number of births (combined), alcohol smoking, family hx of BC, HRT (all and postmenopausal women) and OC use (premenopausal women)

**Table 8:** Randomized controlled trials in adulthood of diet and breast density.

Author, year	Study population, (*n*)	Design	Age	Foods/nutrients of interest	Dietary assessment	Outcome	Major significant results	Adjustments
Bertone-Johnson et al. 2012 [20] (also in Table 3)	WHI Ca + D trial *n* = 330 postmenopausal women with low BD (8.4% ± 10.2%) (US)	RCT	I and C, respectively: 61.8 y, 62.0 y	Daily supplementation of 1,000 mg of Ca and 400 IU of vitamin D (1 y)	122-item FFQ	PBD: (mammogram: computer-assisted method)	Null	Subgroup analyses: age, race/ethnicity, total vitamin D, HT treatment, Gail risk score, BMI, region of residence, category of mammogram density at baseline.

Boyd et al. 1997 [35]	≥50% PBD *n* = 817 (Canada)	RCT	I and C, respectively: 46.5 y, 45.9 y	Low-fat, high-CHO diet (2 y)	3-day food records	AD, PBD at baseline and 2 years (mammogram: automated)	Intervention group: BA ↓ by an average 2.4%. The average ↓ in PBD was 6.1%. Control group: BA was ↑ by 0.3% and PBD was ↓ by 2.1%.	Group assignment, age, weight, menopausal status.

Martin et al. 2009 [36]	≥50% PBD (premenopausal at entry, postmenopausal during followup) *n* = 461 (Canada)	RCT	I and C, respectively: 48.7 y, 48.6 y.	Low-fat, high CHO intervention versus control (2 y)	Food records	TB, DA, NDA, PBD (mammogram: computer-assisted method)	Null	Family hx of BC, OC use, HRT, menopausal status, dietary fat

Knight et al. 1999 [37]	Premenopausal at entry and postmenopausal at followup Total: *n* = 78 (Canada)	RCT	I and C, respectively: 49.5 y, 49.2 y.	Low-fat, high CHO intervention versus control (2 y)	3 food records	ADT, PBD at baseline and 2 years (mammogram: automated)	Total fat (median change: 57–31 g/d) was associated with an average 5.61 cm^2^↓ in the ADT. Saturated fat (median change: 21–11 g/d) and was associated with an average 5.54 cm^2^↓ in the ADT and a 3.93% ↓ in PBD. Dietary cholesterol (median change: 229–150 mg/d) was associated in an average 3.27 cm^2^↓ in the ADT and a 3.52% ↓ in PBD.	Total energy, weight change (included in all models); age, family hx, smoking status, parity, ever breast feeding, OC use, age at menarche, age at first birth, PA

Dorgan et al. 2010 [18] (also in Table 2)	DISC Premenopausal women *n* = 182 (US, NH-White)	CS (RCT followup)	25–29 y	Long-term effects of low-fat diet	3–24-hr dietary recalls	PBD and VDT (MRI)	Null	% body fat, age at randomization, age at visit, clinic, BMI-*Z* score, race, education, smoking status, PA at 14–17 years old and separately during the past year, number of full term pregnancies, hormonal contraceptives

**Table 9:** Studies of total energy and adult breast density.

Author, year	Study population, (*n*)	Design	Age	Foods/nutrients of interest	Dietary assessment	Outcome	Major significant results	Adjustments
Haars et al. 2010 [12] (also in Table 1 and 2)	DOM-project, The Netherlands *n* = 535 (The Netherlands)	CS	>18 y/53 y	Short-term energy restriction	Described in Table 1	BS, DT, NDT, PBD: (mammogram; visual observation)	Null	Age at examination, parity, menopausal status, BMI

Sala et al. 2000 [29] (also in Tables 4 and 5)	EPIC-Norfolk Cases: P2/DY Controls: N1/P1 (*n* = 203 cases & *n* = 203 controls) (UK)	NCC	Cases & Controls: 59 y	Total energy	7-day food record	Wolfe Patterns: (High Risk: P2 & DY; Low Risk: N1 & P1)	All women: total energy**: **OR_*T*3 versus *T*1_ = 1.79, 95% CI: 1.09–2.91) Postmenopausal women: total energy: (OR_*T*3 versus *T*1_ = 2.27, 1.20–4.26)	Unadjusted

Mishra et al. 2011 [13] (also in Tables 1 and 6)	BBC *n* = 700 (England)	PC	36, 43 y/51 y (“habitual adult” dietary patterns)	Total energy	5-day food records	PBD, ADT, ANDT (mammogram; Cumulus)	All women: energy: PBD: Per SD 0.12 (95% CI: 0.01, 0.23) ADT: Per SD: 0.12 (95% CI: 0.00–0.25).	Mammographic view, age at mammogram, BMI at 53, age at menarche, menopausal status at the time of mammography, HT use, parity, smoking status, PA, social class, other three dietary patterns, energy intake.

PC: prospective cohort; CS: cross-sectional; CC: case control; NCC: nested case-control; RCT: randomized controlled trial; I: intervention; C: control; BS: breast size; PBD: percent breast density; VDT: volume of dense tissue; ADT: area of dense tissue; ANDT: area of non-dense tissue; DT: dense tissue; NDT: non-dense tissue; TDA: total dense area; BMI: body mass index; HRT: hormone replacement therapy; MBCFSC: minnesota breast cancer family study cohort; BBC: british birth cohort; MDAS-WHI: mammogram density ancillary study-women's health initiative; WHI CaD: women's health initiative calcium and vitamin d trial; DOM-Project: diagnostisch onderzoek mammacarcinoom-project; EPIC: european investigation into cancer and nutrition; NBCSP: norwegian breast cancer screening program; CNBSS: canadian national breast screening study; DISC: dietary intervention study in children; BEAN: the breast, estrogens, and nutrition study; MEC: the multiethnic cohort; CHO: carbohydrate; MUFA: monounsaturated fatty acids; PUFA; polyunsaturated fatty acids; n-6-FA: omega 6 fatty acids; BC: Breast Cancer; MVMM: multivitamin/multimineral supplement; NH-White: non-hispanic white; OR: odds ratio; FFQ: food frequency questionnaire; BI-RADS: breast imaging-reporting and data system; MDS: mediterranean diet score; WHR: waist-to-hip ratio; PA: physical activity; ER Status: estrogen receptor status; OC: oral contraceptive; Hx: history; MV: multivitamin.

**Table 10:** Summary of nutrient relationships with breast density and their proposed mechanisms.

Nutrients that are associated with an ↑ in breast density (absolute density or % breast density)	
*Premenopausal women: *	*mechanism of action (*i.e.,* IGF/IGFBP/E2/ROS) *
Higher intakes of	
Total fat	↑↓IGF, ↓IGFBP, May ↑Estrogen
SFA (?)	↓IGFBP, ↑IGF, May ↑Estrogen
MUFAs	↓IGFBP, ↑IGF, May ↑Estrogen
n-6 FA	↓IGFBP, ↑IGF, May ↑Estrogen
PUFA	↑IGF, ↓IGFBP, May ↑Estrogen
PUFA : SFA	↑IGF, ↓IGFBP, May ↑Estrogen (?)
Vitamin C (supplemental)	?
Wine	↑Estrogen metabolites, ↑Estrogen responsiveness, ↓SHBG, ↑IGF, ↓IGFBP ↑Oxidative stress
Overall alcohol consumption	↑Estrogen metabolites, ↑Estrogen responsiveness, ↓SHBG, ↑IGF, ↓IGFBP ↑Oxidative stress
MVMM supplements	MAY ↑IGF, ↑IGFBP
Total energy (excess consumed in midlife may affect densities in later life or restriction early in life)	↑Estrogen, ↑IGF availability, ↑DNA replication rate & ↓apoptosis
Lower intakes of	
Carbohydrates	↑IGF (Need to distinguish between whole v. refined, many studies do not do this)
Fiber	↓Oxidative stress (?), may ↑SHBG, ↑IGFBP
Carotene	↓Oxidative stress (?), ↑IGFBP
Calcium	Ameliorates IGF action & enhances IGFBP action (see paper in review), ↑IGF (?)
Protein	Veg Pro = ↑IGFBP
	Total Pro = ↑IGF
Total fat	↓IGFBP, ↑↓IGF, May ↑Estrogen
Saturated fat	↓IGFBP, ↑IGF, May ↑Estrogen
Vitamin B12 (supplemental)	?
White wine	↑Estrogen metabolites, ↑Estrogen responsiveness, ↓SHBG, ↑IGF, ↓IGFBP ↑Oxidative stress
Meat	↑Oxidative stress
Carbohydrates (?)	↑IGF (Need to distinguish between whole versus refined, many studies do not do this)
Total energy	↑Estrogen, ↑IGF availability, ↑DNA replication rate & ↓apoptosis

**Table 11:** Summary of nutrient relationships with breast density and their proposed mechanisms.

Nutrients that are associated with a ↓ in breast density (absolute density or % breast density)	
*Premenopausal women: *↑intakes of:	*Mechanism of action (*i.e.,* IGF/IGFBP/E2/ROS) *
Calcium	May ameliorate IGF action and enhances IGFBP action, ↑IGF (?)
Vitamin D	May ameliorate IGF action and enhances IGFBP action, breast tissue may be able to locally synthesis 25(OH)D→1,25(OH)2D
SFA (?)	(?)
Total dairy	↑IGF, ↑IGFBP, vitamin D and calcium may negate these effects (VD and Ca have stronger effects when IGF/IGFBP are high)
Cheese consumption	↑IGF, ↑IGFBP, vitamin D and calcium may negate these effects (VD and Ca have stronger effects when IGF/IGFBP are high)
Carbohydrate (?)	↑IGF (need to distinguish between whole v. refined, many studies do not do this)
Red Wine	↓Oxidative stress (?)
MUFA	↓Oxidative stress (?)
Carotenoids	↓Oxidative stress (?), ↑IGFBP
Fiber	↓Oxidative stress (?), may ↑SHBG, ↑IGFBP

SFA: saturated fatty acids; MUFA: monounsaturated fatty acids; n-6 FA: omega-6 fatty acids; PUFA: polyunsaturated fatty acids; MVMM: multivitamin/multimineral supplements; IGF: insulin growth factor; IGFBP: insulin growth factor binding proteins; SHBG: sex hormone binding globulin; VD: vitamin d; Ca: calcium.

## References

[1] DeSantis C, Siegel R, Bandi P (2011). Breast cancer statistics. *CA—A Cancer Journal for Clinicians*.

[2] WCRF/AICR (2007). *Food, Nutrition, Physical Activity, and the Prevention of Cancer: A Global Perspective*.

[3] Colditz GA, Frazier AL (1995). Models of breast cancer show that risk is set by events of early life: prevention efforts must shift focus. *Cancer Epidemiology Biomarkers and Prevention*.

[4] http://www.cancer.org.

[5] Boyd NF, Rommens JM, Vogt K (2005). Mammographic breast density as an intermediate phenotype for breast cancer. *The Lancet Oncology*.

[6] Brisson J, Brisson B, Cote G, Maunsell E, Berube S, Robert J (2000). Tamoxifen and mammographic breast densities. *Cancer Epidemiology Biomarkers and Prevention*.

[7] Freedman M, San Martin J, O’Gorman J (2001). Digitized mammography: a clinical trial of postmenopausal women randomly assigned to receive raloxifene, estrogen, or placebo. *Journal of the National Cancer Institute*.

[8] Greendale GA, Reboussin BA, Slone S, Wasilauskas C, Pike MC, Ursin G (2003). Postmenopausal hormone therapy and change in mammographic density. *Journal of the National Cancer Institute*.

[9] Vachon CM, Kuni CC, Anderson K, Anderson VE, Sellers TA (2000). Association of mammographically defined percent breast density with epidemiologic risk factors for breast cancer (United States). *Cancer Causes and Control*.

[10] Lope V, Perez-Gomez B, Sanchez-Contador C (2012). Obstetric history and mammographic density: a population-based cross-sectional study in Spain (DDM-Spain). *Breast Cancer Research and Treatment*.

[11] Butler LM, Gold EB, Greendale GA (2008). Menstrual and reproductive factors in relation to mammographic density: the Study of Women’s Health Across the Nation (SWAN). *Breast Cancer Research and Treatment*.

[12] Haars G, Van Gils CH, Elias SG, TteLokate M, Van Noord PAH, Peeters PHM (2010). The influence of a period of caloric restriction due to the Dutch Famine on breast density. *International Journal of Cancer*.

[13] Mishra GD, Dos Santos Silva I, McNaughton SA, Stephen A, Kuh D (2011). Energy intake and dietary patterns in childhood and throughout adulthood and mammographic density: results from a British prospective cohort. *Cancer Causes and Control*.

[14] Mishra G, McCormack V, Kuh D, Hardy R, Stephen A, Dos Santos Silva I (2008). Dietary calcium and vitamin D intakes in childhood and throughout adulthood and mammographic density in a British birth cohort. *British Journal of Cancer*.

[15] Sellers TA, Vachon CM, Pankratz VS (2007). Association of childhood and adolescent anthropometric factors, physical activity, and diet with adult mammographic breast density. *American Journal of Epidemiology*.

[16] Tseng M, Olufade TO, Evers KA, Byrne C (2011). Adolescent lifestyle factors and adult breast density in U.S. Chinese immigrant women. *Nutrition and Cancer*.

[17] Vachon CM, Sellers TA, Janney CA (2005). Alcohol intake in adolescence and mammographic density. *International Journal of Cancer*.

[18] Dorgan JF, Liu L, Klifa C (2010). Adolescent diet and subsequent serum hormones, breast density, and bone mineral density in young women: results of the dietary intervention study in children follow-up study. *Cancer Epidemiology Biomarkers and Prevention*.

[19] Bertone-Johnson ER, Chlebowski RT, Manson JE (2010). Dietary vitamin D and calcium intake and mammographic density in postmenopausal women. *Menopause*.

[20] Bertone-Johnson ER, McTiernan A, Thomson CA (2012). Vitamin D and calcium supplementation and one-year change in mammographic density in the women's health initiative calcium and vitamin D trial. *Cancer Epidemiology, Biomarkers & Prevention*.

[21] Bérubé S, Diorio C, Mâsse B (2005). Vitamin D and calcium intakes from food or supplements and mammographic breast density. *Cancer Epidemiology Biomarkers and Prevention*.

[22] Bérubé S, Diorio C, Verhoek-Oftedahl W, Brisson J (2004). Vitamin D, calcium, and mammographic breast densities. *Cancer Epidemiology Biomarkers and Prevention*.

[23] Diorio C, Bérubé S, Byrne C (2006). Influence of insulin-like growth factors on the strength of the relation of vitamin D and calcium intakes to mammographic breast density. *Cancer Research*.

[24] Masala G, Ambrogetti D, Assedi M, Giorgi D, Del Turco MR, Palli D (2006). Dietary and lifestyle determinants of mammographic breast density. A longitudinal study in a Mediterranean population. *International Journal of Cancer*.

[25] Nordevang E, Azavedo E, Svane G, Nilsson B, Holm LE (1993). Dietary habits and mammographic patterns in patients with breast cancer. *Breast Cancer Research and Treatment*.

[26] Knight JA, Vachon CM, Vierkant RA, Vieth R, Cerhan JR, Sellers TA (2006). No association between 25-hydroxyvitamin D and mammographic density. *Cancer Epidemiology Biomarkers and Prevention*.

[27] Tseng M, Byrne C, Evers KA, Daly MB (2007). Dietary intake and breast density in high-risk women: a cross-sectional study. *Breast Cancer Research*.

[28] Maskarinec G, Takata Y, Pagano I, Lurie G, Wilkens LR, Kolonel LN (2006). Alcohol consumption and mammographic density in a multiethnic population. *International Journal of Cancer*.

[29] Sala E, Warren R, Duffy S, Welch A, Luben R, Day N (2000). High risk mammographic parenchymal patterns and diet: a case-control study. *British Journal of Cancer*.

[30] Brisson J, Verreault R, Morrison AS, Tennina S, Meyer F (1989). Diet, mammographic features of breast tissue, and breast cancer risk. *American Journal of Epidemiology*.

[31] Nagata C, Matsubara T, Fujita H (2005). Associations of mammographic density with dietary factors in Japanese women. *Cancer Epidemiology Biomarkers and Prevention*.

[32] Qureshi SA, Couto E, Hilsen M (2011). Mammographic density and intake of selected nutrients and vitamins in Norwegian women. *Nutrition and Cancer*.

[33] Tseng M, Vierkant RA, Kushi LH, Sellers TA, Vachon CM (2008). Dietary patterns and breast density in the Minnesota Breast Cancer Family Study. *Cancer Causes and Control*.

[34] Bérubé S, Diorio C, Brisson J (2008). Multivitamin-multimineral supplement use and mammographic breast density. *American Journal of Clinical Nutrition*.

[35] Boyd NF, Greenberg C, Lockwood G (1997). Effects at two years of a low-fat, high-carbohydrate diet on radiologic features of the breast: results from a randomized trial. *Journal of the National Cancer Institute*.

[36] Martin LJ, Greenberg CV, Kriukov V (2009). Effect of a low-fat, high-carbohydrate dietary intervention on change in mammographic density over menopause. *Breast Cancer Research and Treatment*.

[37] Knight JA, Martin LJ, Greenberg CV (1999). Macronutrient intake and change in mammographic density at menopause: results from a randomized trial. *Cancer Epidemiology Biomarkers and Prevention*.

[38] Klifa C, Carballido-Gamio J, Wilmes L (2010). Magnetic resonance imaging for secondary assessment of breast density in a high-risk cohort. *Magnetic Resonance Imaging*.

[39] Balleyguier C, Ayadi S, Van Nguyen K, Vanel D, Dromain C, Sigal R (2007). BIRADS classification in mammography. *European Journal of Radiology*.

[40] Wolfe JN (1976). Breast patterns as an index of risk for developing breast cancer. *American Journal of Roentgenology*.

[41] Gram IT, Funkhouser E, Tabár L (1997). The Tabar classification of mammographic parenchymal patterns. *European Journal of Radiology*.

[42] Byng JW, Yaffe MJ, Jong RA (1998). Analysis of mammographic density and breast cancer risk from digitized mammograms. *Radiographics*.

[43] Rutter CM, Mandelson MT, Laya MB, Taplin S, Seger (2001). Changes in breast density associated with initiation, discontinuation, and continuing use of hormone replacement therapy. *JAMA*.

[44] Stone J, Warren RML, Pinney E, Warwick J, Cuzick J (2009). Determinants of percentage and area measures of mammographic density. *American Journal of Epidemiology*.

[45] Boyd NF, Dite GS, Stone J (2002). Heritability of mammographic density, a risk factor for breast cancer. *The New England Journal of Medicine*.

[46] Ruder EH, Dorgan JF, Kranz S, Kris-Etherton PM, Hartman TJ (2008). Examining breast cancer growth and lifestyle risk factors: early life, childhood, and adolescence. *Clinical Breast Cancer*.

[47] Cerhan JR, Sellers TA, Janney CA, Pankratz VS, Brandt KR, Vachon CM (2005). Prenatal and perinatal correlates of adult mammographic breast density. *Cancer Epidemiology Biomarkers and Prevention*.

[48] Willett WC (1998). *Nutritional Epidemiology*.

[49] Van Noord PAH (2004). Breast cancer and the brain: a neurodevelopmental hypothesis to explain the opposing effects of caloric deprivation during the Dutch Famine of 1944-1945 on breast cancer and its risk factors. *Journal of Nutrition*.

[50] Diorio C, Pollak M, Byrne C (2005). Insulin-like growth factor-I, IGF-binding protein-3, and mammographic breast density. *Cancer Epidemiology Biomarkers and Prevention*.

[51] Vachon CM, Kushi LH, Cerhan JR, Kuni CC, Sellers TA (2000). Association of diet and mammographic breast density in the Minnesota breast cancer family cohort. *Cancer Epidemiology Biomarkers and Prevention*.

[52] Singletary KW, Gapstur SM (2001). Alcohol and breast cancer: review of epidemiologic and experimental evidence and potential mechanisms. *JAMA*.

[53] Dumitrescu RG, Shields PG (2005). The etiology of alcohol-induced breast cancer. *Alcohol*.

[54] Vrieling A, Voskuil DW, Mesquita HBBD (2004). Dietary determinants of circulating insulin-like growth factor (IGF)-I and IGF binding proteins 1, -2 and -3 in women in the Netherlands. *Cancer Causes and Control*.

[55] Maskarinec G, Verheus M, Tice JA (2010). Epidemiologic studies of isoflavones & mammographic density. *Nutrients*.

[56] Lipkin M, Newmark HL (1999). Vitamin D, calcium and prevention of breast cancer: a review. *Journal of the American College of Nutrition*.

[57] Welsh J (2007). Targets of vitamin D receptor signaling in the mammary gland. *Journal of Bone and Mineral Research*.

[58] Cuzick J, Warwick J, Pinney E, Warren RML, Duffy SW (2004). Tamoxifen and breast density in women at increased risk of breast cancer. *Journal of the National Cancer Institute*.

[59] Brisson J, Bérubé S, Diorio C, Sinotte M, Pollak M, Mâsse B (2007). Synchronized seasonal variations of mammographic breast density and plasma 25-hydroxyvitamin D. *Cancer Epidemiology Biomarkers and Prevention*.

[60] Holick MF (2006). Vitamin D: its role in cancer prevention and treatment. *Progress in Biophysics and Molecular Biology*.

[61] Byrne C, Colditz GA, Willet WC, Speizer FE, Pollak M, Hankinson SE (2000). Plasma insulin-like growth factor (IGF) I, IGF-binding protein 3, and mammographic density. *Cancer Research*.

[62] Harvey JA, Bovbjerg VE (2004). Quantitative assessment of mammographic breast density: relationship with breast cancer risk. *Radiology*.

[63] Boyd NF, Connelly P, Byng J (1995). Plasma lipids, lipoproteins, and mammographic densities. *Cancer Epidemiology Biomarkers and Prevention*.

[64] Hu FB (2002). Dietary pattern analysis: a new direction in nutritional epidemiology. *Current Opinion in Lipidology*.

[65] Butler LM, Gold EB, Conroy SM (2010). Active, but not passive cigarette smoking was inversely associated with mammographic density. *Cancer Causes and Control*.

[66] Tseng M, Sellers TA, Vierkant RA, Kushi LH, Vachon CM (2008). Mediterranean diet and breast density in the Minnesota breast cancer family study. *Nutrition and Cancer*.

[67] Hooper L, Madhavan G, Tice JA, Leinster SJ, Cassidy A (2010). Effects of isoflavones on breast density in pre-and post-menopausal women: a systematic review and meta-analysis of randomized controlled trials. *Human Reproduction Update*.

[68] Chan S, Su MY, Lei FJ (2011). Menstrual cycle-related fluctuations in breast density measured by using three-dimensional MR imaging. *Radiology*.

[69] White E, Velentgas P, Mandelson MT (1998). Variation in mammographic breast density by time in menstrual cycle among women aged 40-49 years. *Journal of the National Cancer Institute*.

[70] Morrow M, Chatterton RT, Rademaker AW (2010). A prospective study of variability in mammographic density during the menstrual cycle. *Breast Cancer Research and Treatment*.

[71] Buist DSM, Aiello EJ, Miglioretti DL, White E (2006). Mammographic breast density, dense area, and breast area differences by phase in the menstrual cycle. *Cancer Epidemiology Biomarkers and Prevention*.

